# Learning curve of laparoscopic Kasai portoenterostomy for biliary atresia: report of 100 cases

**DOI:** 10.1186/s12893-018-0443-y

**Published:** 2018-11-26

**Authors:** Yi Ji, Kaiying Yang, Xuepeng Zhang, Siyuan Chen, Zhicheng Xu

**Affiliations:** 1 Department of Pediatric Surgery, West China Hospital of Sichuan University, Chengdu, Sichuan, #37 Guo-Xue-Xiang, Chengdu, 610041 China; 2 Pediatric Intensive Care Unit, Department of Critical Care Medicine, West China Hospital of Sichuan University, Chengdu, 610041 China

**Keywords:** Biliary atresia, Portoenterostomy, Laparoscopy, Learning curve

## Abstract

**Background:**

Laparoscopic Kasai portoenterostomy (LKPE) is performed for biliary atresia (BA). As LKPE is a technically demanding operation, a learning curve should be defined to guide training. The aim of this study was to identify the learning curve of LKPE for BA.

**Methods:**

Metrics of perioperative safety and efficiency for 100 cases of LKPE were evaluated. Outcomes were followed to 67.2 ± 12.1 months. Cumulative sum (CUSUM) analysis was used to identify inflexion point corresponding to the learning curve. Outcome measures included operative time (ORT), rate of clearance of jaundice (CJ) and survival with native liver (SNL).

**Results:**

Between May 2009 and May 2013, 100 consecutive patients with BA underwent LKPE. The rate of conversion from LKPE to open Kasai portoenterostomy (OKPE), intraoperative transfusion and any perioperative complications was 11, 26 and 16%, respectively. There was no perioperative mortality. The CUSUM analysis revealed a learning curve of 50 for LKPE. Precipitous ORT reductions from an initial mean operative time of 316.3 min that was observed in the first 50 to 232.2 min of the late 50 cases (*P* < 0.01)*.* Subsequently, cases 1 to 50 were considered ‘early experience’, whereas cases 51 and higher were considered as ‘late experience’ for statistical analysis. The rate of CJ and SNL was significantly higher after the early 50 cases (*P* < 0.05). In contrast, the rate of intraoperative transfusion, the median time of oral feeding initiated after operation, and the length of hospital stay was not different between the both groups (*P* > 0.05).

**Conclusions:**

In this experience, improved perioperative and postoperative parameters for LKPE were observed in the last 50 patients when compared with the first 50 patients. The dedicated training is likely to contribute to significantly shorter learning curves in future adopters.

## Background

Laparoscopic Kasai portoenterostomy (LKPE) has first been applied to biliary atresia (BA) by Esteves in 2002 [1]. Previously, LKPE had not been showed to be as efficacious as the open Kasai portoenterostomy (OKPE) [2–4]. However, recent reports revealed that the 3- and 5-year survivals with native liver (SNL) after LKPE were not inferior to that after OKPE [5, 6]. Similar to other laparoscopic procedures, a learning curve may exist for LKPE, which, if identified, may allow new adopters to improve their insight. For LKPE, the learning curve may entail the mastery of key points of this specific procedure. These key points include optimal port placement, the full exposure of porta hepatis, the appropriate level of resection of fibrous cone, the demanding technique of portoenterostomy, the development of close coordination between the console surgeon and bedside assistant, and the overcoming of the loss of tactile feedback.

The present paper reports the clinical data of the first 100 cases of LKPE, which were accomplished at our hospital, with the purpose of discerning major inflexion points and landmarks in the optimization of perioperative outcomes.

## Methods

### Design and study population

The first 100 consecutive cases of LKPE were reviewed retrospectively. All operations were carried out by the same surgical team with substantial laparoscopic experiences at West China Hospital of Sichuan University between May 2009 and May 2013. Approval was obtained from the West China Hospital of Sichuan University Institutional Review Board. All procedures followed the research protocols approved by Sichuan University and West China Hospital of Sichuan University and was conducted according to the Declaration of Helsinki. Written informed consent was provided by the patients’ parents for their clinical records to be used in this study.

### Operative technique

The operative technique of LKPE has already been described in a previous study from our group [7]. Briefly, a percutaneous suture was used to snare the round and falciform ligament and retract the liver. Afterward, another two percutaneous transhepatic sutures were introduced individually into the left lobe and right lobe for better hilar exposure. The atretic gallbladder and cystic duct were dissected free from the gall bladder fossa. The distal part of the extrahepatic duct was divided behind the duodenum with 5 mm monopolar hook. After the base of the fibrous cone was reached, the fibrous cord was transected carefully with laparoscopic scissors. The level of resection of fibrous cone was depended on the appearance of bile juice secreted at the fibrous stump. Bleeding from the fibrous remains of portal plate was controlled by direct pressure with moist gauze. Next, a 25-cm Roux-en-Y limb was fashioned and delivered to the hilum through a retro-colic path after the pneumoperitoneum was re-established.

### Definitions and postoperative management

ORT was calculated as the length of time between skin incision and closure. Perioperative complications were complications that occurred during the perioperative period. Theses perioperative complications included wound infection, umbilical hernia, displacement of peritoneal drainage, omentum prolapsed through the trocar wound, respiratory infection, intestinal anastomotic fistula, adhesive intestinal obstruction, etc. Clearance of jaundice (CJ) or jaundice free was defined as the total bilirubin level < 1.2 mg/dL. Early CJ or jaundice free was defined as the total bilirubin level < 1.2 mg/dL within 6 months postoperatively. Incidence of cholangitis was defined as elevated serum bilirubin (> 2.5 mg/dL), leukocytosis with left shift, and normal to acholic stools in a febrile patient (> 38.0 °C).

Steroids, intravenous antibiotics and ursodeoxycholic acid were prescribed to all patients. Methylprednisolone was administered intravenously one day postoperatively at a dose of 1 mg/kg/day for at least 2 weeks. The intravenous antibiotics were administered postoperatively for 2–4 weeks. Patients were followed up regularly until the end of the study.

### Statistical analysis

The software applied for statistical calculation was IBM SPSS 22.0 for windows 10.0 (SPSS Inc., Chicago, IL, USA). In addition to ORT, the rate of CJ, survival with native liver (SNL) and other demographic data were also analyzed. The differences of SNL among the two groups were analyzed by the Kaplan–Meier Method with endpoints of death or liver transplantation and compared by using the log-rank test. Student’s *t*-test and chi-squared were used to analyze continuous variables and qualitative variables, respectively. *P*-values less than 0.05 were considered to be statistically significant.

### Cumulative sum analysis of operative time

Cumulative sum (CUSUM) is the accumulated total difference between each data point and the mean of all data points for a particular metric. CUSUM analysis was well suited to and widely employed in the assessment of new technical skills [8–10]. CUSUM analysis was performed starting with the earliest surgical date. All patients were ordered chronologically. The difference between ORT of each of the 100 cases and the mean ORT of all cases (M-ORT) was then obtained. The CUSUM of ORT was obtained by adding up the calculated difference from the overall mean, starting with the first case to the next cumulatively. If the ORT for a case is more than M-ORT, the addition to the running value of CUSUM of ORT is a positive number (upwards slope on the graph). Conversely, it is a negative number if the ORT for a case is less than M-ORT (downwards slope). This cumulative process is sustained until CUSUM of ORT for the last case is calculated as zero. This allows for a graphical representation of the learning curve, which can be calculated and plotted with the software of Matlab 2016a for Windows 10.0 (Mathworks, Natick, MA).

## Results

### Perioperative outcomes for the entire cohort

Table 1 demonstrates perioperative parameters for the entire cohort of patients (*n* = 100). All included cases were non-syndromic BA. The Type III, II and cyst were 97, 2 and 1%, respectively. A total of 64.0% were female. The median age of the patients was 82.8 days. The median weight was 4.4 kg. The median time of ORT, oral feeding initiated after operation and length of hospital stay was 274 min, 4.2 days and 23.6 days, respectively. The rate of conversion from LKPE to OKPE, intraoperative transfusion and any perioperative complications was 11, 26 and 16%. No patients died during the perioperative period. After a median time of 67.2 months follow-up, the rates of cholangitis and CJ were 66 and 62%, respectively. The 1-year, 3-year and 5-year SNL rate were 66, 49 and 34%, respectively.

**Table 1:** Demographic data of patients of two groups with BA

	Total *n* = 100	Group A *n* = 50	Group B *n* = 50	*P*
Age, (d)	82.8 ± 8.4	81.4 ± 6.4	84.5 ± 9.9	0.51
Female, n (%)	64/100 (64%)	31/50 (62%)	33/50 (66%)	0.68
Weight, (kg)	4.4 ± 0.4	4.4 ± 0.3	4.4 ± 0.4	0.49
Type (III), n (%)	97/100 (97%)	49/50 (98%)	48/50 (96%)	0.60
Type (II), n (%)	2/100 (2%)	1/50 (2%)	1/50 (2%)	1.0
Type (cyst), n (%)	1/100 (1%)	0	1/60 (1.7%)	–
ORT (min)	274.2 ± 55.4	316.3 ± 41.6	232.2 ± 29.3	< 0.01
Conversion rate, n (%)	11/100 (11%)	10/50 (20%)	1/50 (2%)	< 0.01
Intraoperative transfusion, n (%)	26/100 (26%)	16/50 (32%)	10/50 (20%)	0.17
Any perioperative complications, n (%)	16/100 (16%)	12/50 (24%)	4/50 (8%)	< 0.05
Mortality, n (%)	0	0	0	1.0
Time of oral feeding initiated after operation (d)	4.2 ± 1.5	3.7 ± 1.3	4.6 ± 1.6	0.43
Length of hospital stay (d)	23.6 ± 4.3	22.6 ± 3.8	24.5 ± 4.5	0.23
Time of follow-up(month)	67.2 ± 12.1	76.7 ± 8.9	57.5 ± 5.5	< 0.05
Cholangitis rate, n (%)^a^	66/100 (66%)	39/50 (78.0%)	27/50 (54.0%)	< 0.01
CJ rate, n (%)	66/100 (66%)	28/50 (56.0%)	38/50 (74.0%)	< 0.05
1-year SNL rate, n (%)	74/100 (74%)	66.0% (33/50)	82.0% (41/50)	–
3-year SNL rate, n (%)	59/100 (59%)	54.0% (27/50)	64.0% (32/50)	–
5-year SNL rate, n (%)	45/100 (45%)	36.0% (18/50)	54.0% (27/50)	–

^a^Within one year after operation;

*BA*: biliary atresia; *CJ* clearance of jaundice; *ORT* operative time; *SNL* survival with native liver

### Identification of learning curve based on CUSUM of ORT

**Fig. 1:**
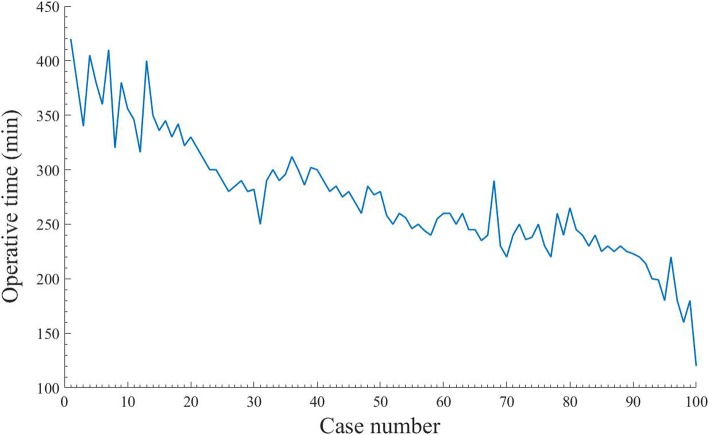
Decreasing variance from the mean operative time (ORT) with the increasing experience in laparoscopic Kasai portoenterostomy (LKPE)

**Fig. 2:**
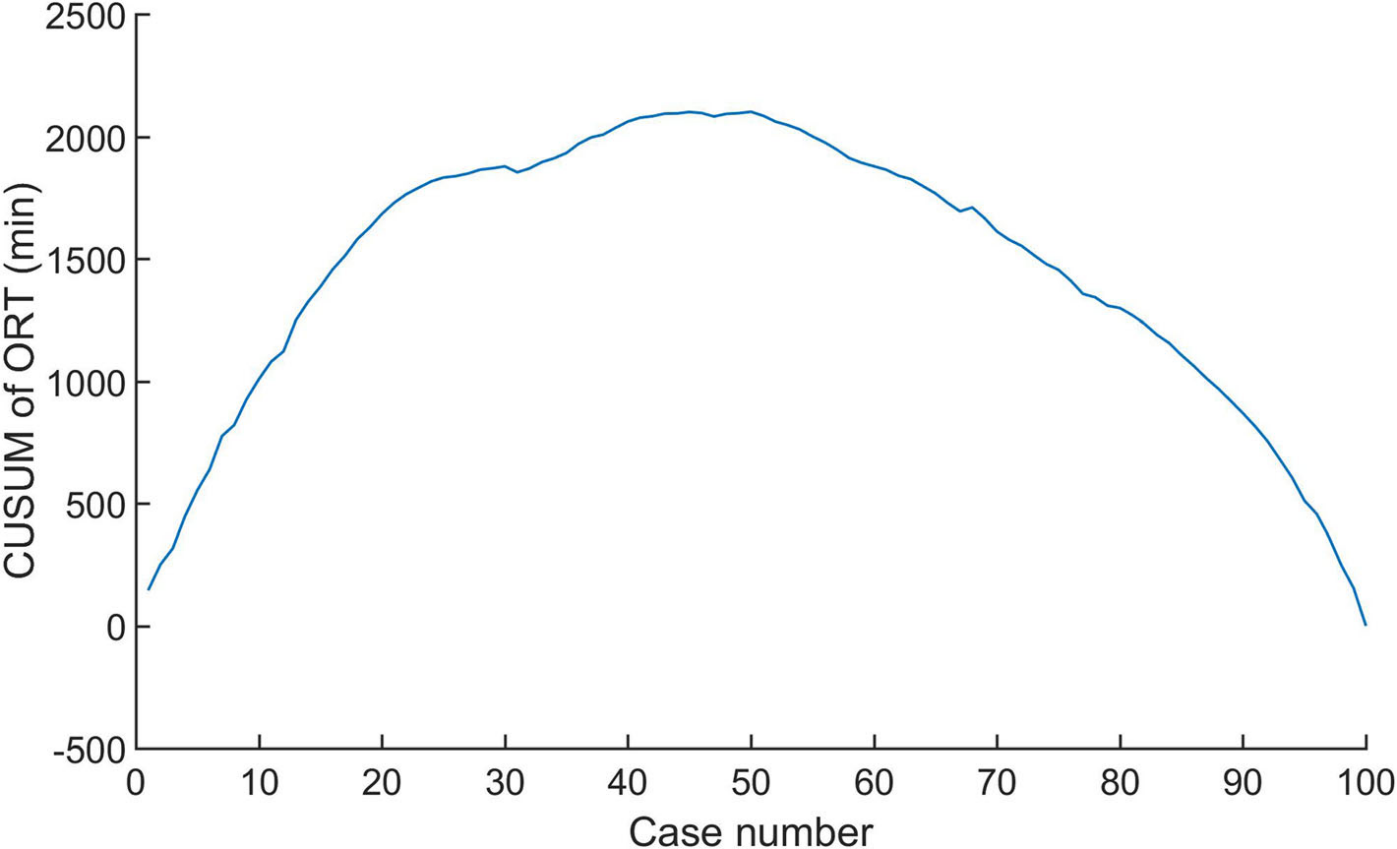
CUSUM analysis of operative time (ORT) shows two distinct phases of the learning curve for laparoscopic Kasai portoenterostomy (LKPE). CUSUM of ORT is plotted on the vertical axis against the respective case number. Phase 1 reveals a sharp rise in CUSUM of ORT before the first 20 cases. Phase 2 displays a significant reduction in CUSUM of ORT after case 20, followed by a very slow increase in CUSUM of ORT with stabilization near case 50. Phase 3 indicates a significant reduction in CUSUMORT after case 50

CUSUM revealed ORT as the one of variables to exhibit improvement across all the patients, whose results of ORT were divided into 10 groups chronologically (Fig. 1). We found that ORT was the only variable to exhibit improvement across the 10 groups (data not shown). A significant reduction in ORT was displayed after the first 50 cases (from 316.3 min to 232.2 min; *P* < 0.01) (Fig. 2). These findings confirmed that the two distinct phases of the learning curve in the first 50 cases, followed by a predominantly downward slope. Patients were stratified into 2 groups of 50 patients, with chronological order defining early and late experience. Furthermore, the ORTs beyond the learning curve continued to improve gradually.

### Perioperative outcomes in relation to the learning curve

**Fig. 3:**
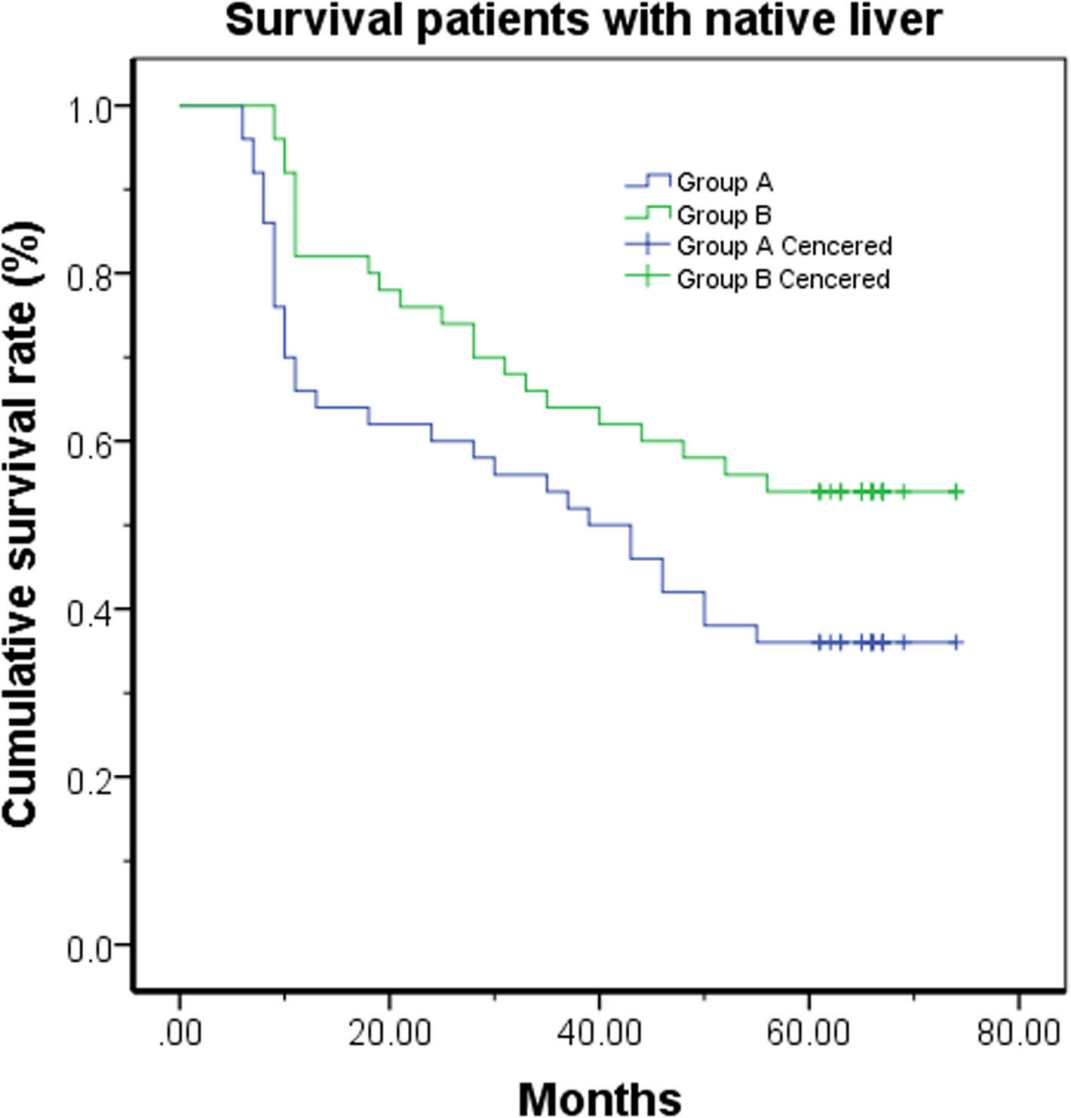
Kaplan–Meier analysis shows that the survival of patients with native livers (SNL) is statistically significant different between Group A and Group B (*P* < 0.05)

Based on the identification of the ORT learning curve of 50 cases, an analysis of perioperative outcomes comparing those of in the learning curve cohort (first 50 cases were assigned to ‘early experience’ or group A), with those in the later experience (post-learning curve cohort: cases 51–100 were assigned to group B) was performed. The two groups were homogeneous with respect to age, female ratio, weight, and type of BA of patients (Table 1). The conversion rate from LKPE to OKPE in group A was much higher than that in group B (*P* < 0.01). The perioperative complications in group A were more common than that in group B (*P* < 0.05). When compared between the both groups, the rate of intraoperative transfusion, the median time of oral feeding initiated after operation, and the length of hospital stay was not different respectively. The postoperative cholangitis in Group B was significant lower than that in Group A (*P* < 0.01). In contrast, the CJ rate in Group B was significant higher than that in Group A (*P* < 0.05). The median time of follow up was 76.7 months in Group A and 57.5 months in Group B, respectively. The Kaplan–Meier method analysis showed that SNL rate in Group A was significant difference when compared with that in Group B (*P* < 0.05) (Fig. 3).

## Discussion

The adaptations of new technologies are challenges particularly in the practice of medicine [11–15]. There is evidence that the effect of experience is clearly cumulative and can be carried over to later performance [11–18]. With the aim to better understanding of surgeons’ performance during our experience with LKPE, we used CUSUM technology, with combination of CJ and SNL rates, to identify the number of cases that are necessary to achieve the competence of LKPE. These efforts could facilitate more effective training and improve the results of LKPE.

The CUSUM technique is widely used to analyze the learning curve for surgical procedure [11, 12] and transforms raw data into running total data deviation from their group mean, enabling investigators to visualize the milestone of learning curve. In this analysis, CUSUM yielded a parabolic curve showing two distinct phases from which correlates of the LKPE learning curve can be assessed. The median ORTs during the first and second phases were 316.3 min and 232.2 min, respectively. The first phase of steep CUSUM of ORT rapidly rise at case 1~ 20, with a relative stabilization at case 20~ 50, can be attributed to increased familiarity with the ‘basics’ of the performance. These include the optimal port placement, the full exposure of porta hepatis, the initial improvement of skills in dissection, the hemostasis, and the portoenterostomy. The phase 2 after case 50 is likely to represent the steep learning curve that reflects the surgeon’s development of ability.

As shown, patients in both groups were similar in terms of demographics, weight, and type of BA, suggesting that minimal selection bias was present to influence our results. The conversion rate from LKPE to OKPE, any perioperative complications rate, and cholangitis rate was 20, 24 and 78% in group A, each of which was significant higher than that observed in group B. In addition, the CJ rate and SNL rate in group A were worse than that in group B. All perioperative results of both groups mentioned above are highly consistent with the learning curve of LKPE, which was plotted by the cumulative calculation of CUSUM of ORT. A possible explanation for these progression observed in the late-experience period is that the surgeons became more comfortable with postoperative care as they gained experience with the operation.

Interestingly, we observed similar rates of rate of intraoperative transfusion. Although perioperative complications rate increased substantially in group B, this finding did not translate into an overall prolonged hospital length of stay. We found that the median time of oral feeding initiated after operation and the length of hospital stay was not different between two groups.

The 50 cases of learning curve in our study was more than that reported by Li et al. [19]. The authors drew the conclusion that without the usage of Cumulative sum analysis for ORT and Kaplan–Meier analysis for SNL. Nevertheless, we agree with Li that LKPE is really a demanding procedure, especially for novices and those in hospitals with low caseload of BA. In addition, the 50 cases of learning curve of LKPE is more than that of laparoscopic procedure for choledochal cyst reported by Diao’s (35 cases) [20] and that of LPCC reported by Zhe’s (37 cases) [21]. The younger age at operation, the poor exposure of porta hepatis, the difficult assessment for level of dissecting biliary fibrous cone, the challenging procedure for portoenterostomy, all of which may contribute to the “long” learning curve of LKPE.

The age at operation from 1 month to 4 months in our study means that the peritoneal space for manipulation of LKPE is limited. Usually, the full exposure of porta hepatis is often difficult, especially during the initial phase of learning curve. We found that the porta hepatis could be totally displayed by suspension of the segment IV only with percutaneous transhepatic suture. Under the magnification of laparoscopy, the view of operative field can be shown clearly and stably on the screen. Furthermore, the proper level of resecting the bilious fibrous cone is hard to be reached under laparoscopy. Our experiences are that the fibrous cone should be dissected between the first bifurcation of portal vein, and gradually pushed deeper until the leakage or oozing of bile-like liquid is found on the surface of the fibrous stump. Therefore, the base of the fibrous cone would be kept intact. When the portoenterostomy is undertaken, the seams of the anastomoses should be kept as far as possible from the surface of the stump. In addition, the rigorous steroids and prophylactic antibiotic treatment for cholangitis after operation is also necessary for achievement for better long-term results.

Although the outcomes of LKPE are not satisfied with OKPE in some reports [6, 22, 23], our recent study revealed that the 3-year and 5 year SNL rates after LKPE were not different compared to those after OKPE. Because BA is a rare disease, the experiences of LKPE are incapable to be cumulated to proficiency in a short time, especially in hospitals or centers with the low caseload of BA. However, the learning curve of LKPE can be shortened by training in the animal experiments, the training devices, such as laparoscopic box and the virtual reality laparoscopic simulator.

Our study has several limitations. Firstly, as a retrospective review, nearly all cases are typeIII non-syndromic BA, which may not fully represent the entire population of BA. Most patients of syndromic BA in our hospital were given up by their parents, which could have an impact on the learning curve of LKPE. Secondly, the learning curve may actually be shorter than 50 cases for surgeons already experienced in some facets of laparoscopic surgery. The staffs with prior laparoscopic experience and training have been able to climb this learning curve much more rapidly, especially that there are many cases of BA performed each year. Thirdly, the present learning curve analysis is based mainly on ORT, CJ and SNL, which probably failed to display constant significant improvements as a result of small sample sizes.

## Conclusions

In conclusion, the current study identifies the learning curve of LKPE to be approximately 50 cases performed by one team of surgeons with some laparoscopic experiences. The learning curve of LKPE is perhaps more pronounced than many other advanced laparoscopic procedures. The mentorship and dedicated training are likely to contribute to the significant shortening of the learning curve of LKPE in future adopters.

## Abbreviations

BA: Biliary atresia
CJ: Clearance of jaundice
CUSUM: Cumulative sum
LKPE: Laparoscopic Kasai portoenterostomy
OKPE: Open Kasai portoenterostomy
ORT: Operative time
SNL: Survival with native liver
